# Neonatal Cholestasis Over Time: Changes in Epidemiology and Outcome in a Cohort of 154 Patients From a Portuguese Tertiary Center

**DOI:** 10.3389/fped.2020.00351

**Published:** 2020-06-30

**Authors:** Ermelinda Santos Silva, Alexandra Almeida, Simão Frutuoso, Esmeralda Martins, Maria João Valente, Alice Santos-Silva, Ana Isabel Lopes

**Affiliations:** ^1^Paediatric Gastroenterology Unit, Centro Materno-Infantil do Norte, Centro Hospitalar Universitário do Porto, Porto, Portugal; ^2^Integrated Master in Medicine, Instituto de Ciências Biomédicas Abel Salazar, Universidade do Porto, Porto, Portugal; ^3^UCIBIO-REQUIMTE, Laboratory of Biochemistry, Faculdade de Farmácia, Universidade do Porto, Porto, Portugal; ^4^Neonatology Unit, Centro Materno-Infantil do Norte, Centro Hospitalar Universitário do Porto, Porto, Portugal; ^5^Metabolic Diseases Reference Center, Centro Materno-Infantil do Norte, Centro Hospitalar Universitário do Porto, Porto, Portugal; ^6^Paediatric Gastroenterology Unit, Hospital Universitário de Santa Maria, Centro Hospitalar Lisboa Norte, Lisbon, Portugal; ^7^Faculdade de Medicina, Universidade de Lisboa, Lisbon, Portugal

**Keywords:** neonatal cholestasis, transient cholestasis, neonatal cholestasis epidemiology, cholestasis risk factors, neonatal cholestasis survival, next generation sequencing panel

## Abstract

**Introduction:** In the last two decades there have been advances in the diagnosis and management of neonatal cholestasis, which may have changed its epidemiology, diagnostic accuracy, outcomes, and survival. Our goal was to characterize these changes over time in our setting.

**Methods:** Retrospective cohort study in a tertiary center, enrolling patients born between January 1985 and October 2019. The cohort was divided into two periods, before (A; *n* = 67) and after (B; *n* = 87) the year 2000; and in two groups, according to patient's outcome (favorable, unfavorable). Overall survival and survival with and without orthotopic liver transplant (OLT) were evaluated in the two periods (A and B) and in different subgroups of underlying entities.

**Results:** We found that the age of cholestasis recognition decreased significantly from period A to period B [median 43 days and 22 days, respectively, (*p* < 0.001)]; the changes in epidemiology were relevant, with a significant decrease in alpha-1-antitrypsin deficiency (*p* < 0.001) and an increase in transient cholestasis (*p* = 0.004). A next-generation sequencing (NGS) panel available since mid-2017 was applied to 13 patients with contributory results in 7, but, so far, only in 2 patients led to conclusive diagnosis of underlying entities. The number of cases of idiopathic cholestasis did not vary significantly. Over time there was no significant change in the outcome (*p* = 0.116). Overall survival and survival without OLT had no significant improvement during the period of observation (in periods A and B, 86 vs. 88%, and 85 vs. 87%, respectively). However, in period B, with OLT we achieved the goal of 100% of survival rate.

**Conclusions:** Our data suggest that transient cholestasis became a very important subset of neonatal cholestasis, requiring specific guidance. The NGS panels can provide important inputs on disease diagnosis but, if applied without strict criteria and expertise, they can open a Pandora's box due to misinterpretation. Despite all the advances in accurate diagnosis and timely management—including early recognition of cholestasis—the improvement in patient outcomes and survival were still not significant.

## Introduction

In the twenty-first century, neonatal cholestasis (NC) remains a major clinical challenge for several reasons. Recognition of NC among jaundiced neonates is delayed in a significant number of cases, often due to the lack awareness of healthcare professionals (1–3). Furthermore, the diagnosis is difficult due to the great diversity of underlying entities, some of them with specific treatment that should be offered in a timely manner to improve prognosis. Finally, morbidity and mortality are still high and many patients survive at the expense of orthotopic liver transplantation (OLT) (4).

Nevertheless, in the last two decades, diagnosis and management has improved, which may have had a significant impact in epidemiology and outcome of NC (5–7). New underlying entities have been added to the long list of etiological causes of NC (3) requiring specialized diagnostic tools and clinical expertise (8). Next-generation sequencing (NGS) technology is a new and appealing diagnostic tool in this field, yet to be incorporated in clinical practice (9). Additionally, the recent advances in understanding the pathophysiology of NC has not yet fully translated into new treatments or prevention strategies (10).

A few studies (11–14) have focused on NC epidemiology and how outcomes have changed over time. A systematic review of 17 studies including a total of 1,692 patients recruited ascertained from 12 countries and 5 continents from 1963 to 2011, focused only in the etiology of NC and was flawed by the inconsistency of diagnostic approach (15).

Moreover, the variations in the terminology used over time and in different centers make it even more difficult to analyse the underlying etiologies. For example, sometimes the term “neonatal hepatitis” is used regardless of existence of a known underlying entity; similar patients may be classified as “transient cholestasis” or as “total parenteral nutrition (TPN) associated cholestasis;” and finally, there is barely no consensus on which cases should be under the broad label of “idiopathic cholestasis.” In addition, the selected studies do not allow us to draw conclusions about the epidemiological trends over time. Outcomes and survival were not systematically assessed.

The aim of this study—the first one in Portugal—was to characterize NC evolution over time, epidemiology, diagnostic accuracy, outcomes, and survival.

## Patients and Methods

### Patients and Study Design

Retrospective cohort study of NC patients diagnosed and treated at the hepatology clinic of a single tertiary hospital, from 1 January 1985 to 31 October 2019. The study was based on the analysis of clinical charts and institutional database records. Patients with incomplete clinical records were excluded.

This study was compliant with the ethical standards of the participating healthcare institution committee [Studies N/REF.ª 2016. 081 (069-DEFI/066-CES) and N/REF.ª 2016. 084 (072-DEFI/069-CES)], and with the 1964 Helsinki declaration and its later amendments or comparable ethical standards.

Patients were referred from institutional departments of Emergency, Neonatology and Pediatrics as well as external institutions (primary and secondary healthcare providers).

We analyzed the cohort according to the period of diagnosis using the year 2000 as a symbolic landmark: period (A) from 1985 to 1999, and period (B) from 2000 to 2019. The cohort was also analyzed according to the outcome (I), unfavorable [death, OLT, advanced chronic liver disease], or (II) favorable (mild chronic liver disease, cure of liver disease). Furthermore, we studied the 7 subgroups of underlying entities of our cohort [biliary diseases, alpha-1-antitrypsin deficiency (A1ATD), infectious diseases, metabolic diseases, transient cholestasis, other diseases, idiopathic cholestasis].

### Clinical Data, Diagnostic Approach and Treatment

#### Patients Were Managed by the Same Nuclear Team Throughout the Years

NC was defined as jaundice with conjugated bilirubin ≥1 mg/dl (and > 20% of total bilirubin, if total bilirubin >5.0 mg/dl), detected in a newborn or infant younger than 4 months old, according to the Guideline of the North American Society for Pediatric Gastroenterology, Hepatology and Nutrition (NASPGHAN) and European Society for Pediatric Gastroenterology, Hepatology and Nutrition (ESPGHAN) (16). For the retrospective analysis, transient cholestasis was defined as the presence of cholestatic jaundice, with known risk factors, in the same age group, and complete and spontaneous normalization of liver function tests within the first 6 months of life (17, 18).

The risk factors for NC included those described by Champion et al. (19), as well as sepsis and asphyxia, among others (20, 21). Signs and symptoms of sepsis were moaning, lethargy, hypotonia, fever, and/or hypothermia. Signs and symptoms of liver failure were coagulopathy not correctable by vitamin K administration, ascites, and hypoglycaemia, in accordance to the Pediatric Acute Liver Failure Study Group (22).

Diagnostic approaches evolved over the years reflecting the evolution of international guidelines and accumulated personal experience (8, 16). Diagnosis of underlying entities was based on biochemical, imaging, histological, and enzymatic tests; molecular studies were also performed when appropriate and available. Diagnosis of biliary atresia was confirmed by per-operatory cholangiography (with simultaneous liver biopsy). Diagnosis of liver disease for A1ATD was based on the serum level of the protein and its phenotype, and on liver histology.

Since 2005 the national newborn screening program (performed between the 3rd and 6th day of life) includes the screening of 24 treatable disorders, namely hypothyroidism, tyrosinemia, argininemia, citrullinemia type II, some organic acidurias and beta-oxidation fatty acids disorders, and cystic fibrosis (23).

In the mid-2017 a customized NGS panel targeting inborn errors of metabolism and genetic cholestatic disorders became available—initially comprised of 54 genes, and currently 95 genes (Table 1). This panel was offered to some patients fulfilling the following criteria: no known underlying entity (liver failure survivors, and/or with evolving chronic liver disease), suspicion of a second underlying entity, and cholestasis evolving transiently without risk factors.

**Table 1:** List of genes included in the next generation sequence (NGS) panel.

	**NGS panel evolution**	
	**Mid-2017 (54 genes)**	**Since the end of 2019 (95 genes)**
**Genes**		
	ABCB11, ABCB4, ACAD9, AKR1D1, ASAH1 ATP8B1, BAAT, BCS1L CC2D2A, CFTR, CLDN1 CYP27A1, CYP7A1, CYP7B1 DCDC2, DGUOK GBA, GBE1, HSD3B7 INVS, JAG1, LIPA MKS1, MPV17, MYO5B NOTCH2, NPC1, NPC2, NPH3, NR1H4 PEX1, PEX10, PEX11B, PEX12, PEX13, PEX14, PEX16, PEX19, PEX2, PEX26, PEX3, PEX5, PEX6, PEX7 PKHD1, POLG, POLG2 RRM2B SERPINA1, SLC25A13 TJP2, TRMU VIPAS39, VPS33B	ABCB11, ABCB4, ABCC2, ABCD3, ABCG5, ABCG8, ADK, AKR1D1, ALDOB, AMACR, ATP7B, ATP8B1, BAAT, BCS1L, CC2D2A, CFTR, CLDN1, COG7, CYP27A1, CYP7A1, CYP7B1 DCDC2, DGUOK, DHCR7, EHHADH, EPHX1, FAH, GALT, GBA, GBE1, GNAS, GPBAR1 HADHA, HAMP, HFE, HFE2, HNF1B, HSD17B4, HSD3B7, IARS, INVS, JAG1, LIPA, MKS1, MPI, MPV17, MVK, MYO5B, NOTCH2, NPC1, NPC2, NPHP1, NPHP3, NPHP4, NR1H4, PEX1, PEX10, PEX11B, PEX12, PEX13, PEX14, PEX16, PEX19, PEX2, PEX26, PEX3, PEX5, PEX6, PEX7, PKHD1, POLG, POLG2, RPGRIP1L, SCP2, SERAC1, SERPINA1, SLC10A1, SLC10A2, SLC25A13, SLC27A5, SLC30A10, SLC40A1, SMPD1, TALDO1, TFR2, TJP2, TMEM216, TMEM67, TRMU, TTC37, UGT1A1, USP53, UTP4, VIPAS39, VPS33B

Patients with biliary atresia were submitted to Kasai porto-enterostomy (PE), performed by a stable and skilled surgical team after January 2000 (24). Patients with A1ATD born after 1994 were treated by ursodeoxycholic acid (UDCA), 15–20 mg/kg/day, bid (25). Metabolic diseases were treated with special diets and specific drugs, according to the state of art, which included OLT in some patients (26, 27). Pediatric OLT was available in our country since January 1994 (one center); before was only available through foreign centers. All patients with chronic cholestasis were managed with supportive therapy in accordance to international guidelines (28).

### Statistical Analysis

Clinical, biochemical, and genetic data were compared by using the following tests, as appropriate: chi-square, Fisher's and Mann-Whitney. Overall survival and survival without OLT were compared between groups using the Kaplan-Meier method and the Log Rank. The significance level used was *P* < 0.05. Statistical analysis was performed using the software Statistical Package for the Social Sciences v. 24.0.

## Results

We analyzed 154 clinical charts of NC patients (6 were excluded due to insufficient data). The descriptive analysis of the cohort is available on Table 2.

**Table 2:** Descriptive analysis of the cohort and comparisons between periods A (before year 2000) and B (after year 2000).

	**Cohort (*****N*** **=** **154)**			**Before year 2000 (A)**			**After year 2000 (B)**			
	***N***	**Median/IQ**	**Range**	***n***	**Median/IQ**	**Range**	***n***	**Median/IQ**	**Range**	*p*-value^*^
1. Age of onset of jaundice (days)	154	2 (2–4)	1–118	67	3 (2–7)	1–80	87	2 (2–3)	1–118	**0.015**
2. Age of cholestasis recognition (days)	153	30 (8–54)	1–165	66	43 (21–72)	2–165	87	22 (6–42)	1–124	** <0.001**
3. Age of admission to the tertiary center (days)	153	54 (30–75)	3–192	66	61 (33–79)	3–165	87	45 (30–69)	7–192	0.072
4. Age of diagnosis of underlying entity (days)	154	65 (42–97)	4–2,520	67	75 (45–97)	4–2,520	87	61 (42–100)	10–270	0.371
Interval between 1 and 2 (days)	153	22 (6–41)	0–163	66	31 (15–58)	0–163	87	16 (3–33)	0–75	** <0.001**
Interval between 2 and 3 (days)	153	8 (1–27)	0–143	66	6 (0–14)	0–99	87	12 (1–49)	0–143	**0.017**
Interval between 3 and 4 (days)	152	7 (4–14)	0–2,458	65	6 (5–11)	0–2,458	87	7 (4–17)	0–221	0.355
Gestational age (weeks)	144	39 (36–40)	26–42	63	40 (39–40)	30–42	81	37 (34–39)	26–41	** <0.001**
Birth weight (grams)	148	2,858 (2,200–3,280)	570–4,400	66	3,045 (2,400–3,425)	1,000–4,400	82	2,615 (1,626–3,070)	570–4,000	**0.002**
Age at Kasai surgery (days)	24	62 (42–72)	31–128	9	84 (64–110)	42–128	15	47 (39–63)	31–69	**0.002**
Age at OLT (months)	28	24 (13–69)	8–132	18	30 (18–90)	8–132	10	16 (12–46)	8–81	0.088
Age of death (months)	18	9 (4–23)	2–32	9	14 (5–27)	2–32	9	9 (2–19)	2–25	0.248
Follow-up (months), maximum 216 (18 years)	154	118 (21–216)	2–216	67	216 (72–216)	2–216	87	60 (18–126)	2–216	** <0.001**

* *Mann-Whitney test; IQ, interquartile; OLT, orthotopic liver transplantation*.

*The bold values correspond to results with significative differences*.

The male gender showed a higher prevalence (*n* = 92; 60%). Parental consanguinity was present in 9 patients (6%) and 11 (7%) had siblings affected by related clinical conditions.

A comparison between period A (before year 2000) and period B (after year 2000) is also available in Table 2. We analyzed 67 patients in period A and 87 patients in period B. The median follow-up was, as expectable, significantly longer in period A (*p* < 0.001].

### Cholestasis Recognition Over Time

The median age of onset of jaundice, cholestasis recognition, and admission to the tertiary center, before and after year 2000 are described and compared in Table 2.

We verified that the age of cholestasis recognition decreased significantly after the year 2000 [median = 43 days (IQ: 21–72) vs. 22 days (IQ: 6–42), *p* < 0.001], while the time interval between the cholestasis recognition and admission to the tertiary center increased significantly [median 6 days (IQ: 0–14) vs. 12 days (IQ: 1–49), *p* = 0.017]; however, the age of admission to the tertiary center decreased in period B, but non significantly.

### Role of Risk Factors

Risk factors for developing neonatal cholestasis were present in 45% (*n* = 69/85) of the cohort. Gestational age (*p* < 0.001) and birth weight (*p* = 0.002) were both significantly lower in period B.

All patients from the subgroup of transient cholestasis had at least one risk factor, and the same was observed in 35% (*n* = 45/85) of all other patients (*p* < 0.001) (Table 3).

**Table 3:** Risk factors for neonatal cholestasis: comparison between transient cholestasis and other patients.

	**Cohort (*****N*** **=** **154)**		**Transient cholestasis**		**Other patients**		
	***N***	**(%)**	***N***	**(%)**	***n***	**(%)**	*p*-value^*^
**RISK FACTORS**							
**Gestational age**							** <0.001**
Term	114	(74)	4	(17)	110	(85)	
Preterm ≥34 weeks	16	(10)	4	(17)	12	(9)	
Preterm <34 weeks	24	(16)	16	(17)	8	(6)	
**Birth weight**							** <0.001**
Normal	93	(62)	4	(17)	89	(69)	
Low	34	(23)	8	(33)	26	(20)	
Very low	8	(5)	3	(13)	5	(4)	
Extremely low	10	(7)	9	(38)	1	(0.8)	
Big	6	(4)	0	0	6	(5)	
Missings					3		
**Birth weight vs. gestational age**							0.125
Adequate	110	(71)	13	(54)	97	(75)	
Small	40	(26)	10	(42)	30	(23)	
Big	4	(3)	1	(4)	3	(2)	
**TPN** **>7 days**					130		** <0.001**
Yes	38	(25)	21	(88)	17	(13)	
**Infection/sepsis**							** <0.001**
Yes	56	(36)	21	(88)	35	(27)	
**Surgery**							**0.045**
Yes	7	(4)	3	(13)	4	(3)	
Thoracic	1	(0.6)	1	(4)	0	0	
Abdominal	5	(3)	2	(8)	3	(2)	
Pelvic	0	(0)	0	0	0	0	
Brain	1	(0.6)	0	0	1	(0.8)	
**Invasive ventilation**							** <0.001**
Yes	25	(16)	16	(67)	9	(7)	
**Hemodynamic instability**							** <0.001**
Yes	16	(10)	8	(33)	8	(6)	
**Number of RF (19)**							** <0.001**
0	85	(55)	0	0	85	(65)	
1	40	(26)	6	(25)	34	(26)	
2	20	(13)	10	(42)	10	(8)	
3	9	(6)	8	(33)	1	(0.8)	
4	0	(0)	0	0	0	0	
**One OR more RF**							** <0.001**
Yes	69	(45)	24	(100)	45	(35)	

* *Chi-square test; RF, Risk factors; TPN, total parenteral nutrition*.

*The bold values correspond to results with significative differences*.

The presence of one or more risk factors was significantly less frequent in patients with biliary atresia in comparison to other patients, even when patients with transient cholestasis were excluded from analysis (*p* = 0.008), which is in contrast to what was observed with A1ATD patients (*p* = 0.541).

### Diagnosis of Underlying Entities Over Time

**Figure 1:**
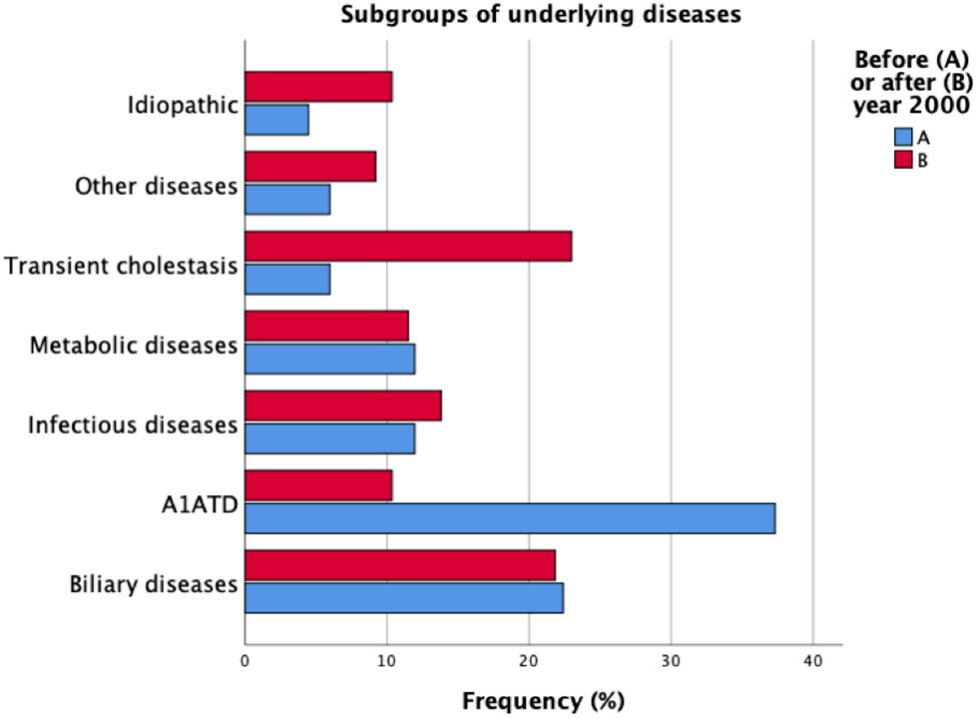
Underlying entities in the cohort of patients with neonatal cholestasis (*N* = 154). Comparison between periods of time A and B.

A1ATD (*n* = 34, 22%) and biliary atresia (*n* = 24, 16%) were the most prevalent underlying entities. Transient cholestasis was one of the major sub-groups of patients (*n* = 24, 16%). Over the years, epidemiological trends underwent significant changes in our cohort (Figure 1).

#### Biliary Atresia and A1ATD—The Old Players

While the frequency of biliary atresia remained stable, A1ATD has significantly lost prominence over the years [period A: *n* = 25 (37%); period B: *n* = 9 (10%), *p* < 0.001]. The A1AT phenotypes were ZZ (*n* = 29), SZ (*n* = 3), M1Z (*n* = 1), and FZ (*n* = 1). One of the SZ patients had concomitantly biliary atresia.

#### Transient Cholestasis—The New Kid on the Block

From 33 patients referred with transient cholestasis, only 24 remained with this diagnosis after investigation; in 9 of these patients we identified post-natal Cytomegalovirus (CMV) infection, A1ATD, choledochal cyst, and chromosomal disorders. Transient cholestasis emerged as the major subgroup of patients in period B [*n* = 4 (6%) vs. *n* = 20 (23%), *p* = 0.004].

#### Idiopathic Neonatal Cholestasis—What's Been Left in the Bag

Idiopathic neonatal cholestasis accounted for 8% (*n* = 12) of all patients, and the difference over time was not significant.

### Contribution of Molecular Studies

Molecular studies were more commonly used in period B (*n* = 21; 24%) than in period A (*n* = 6; 9%) (*p* = 0.014). Time lag between admission and etiological diagnosis was significantly longer than in patients who did not undergo genetic testing [23 days (IQ: 3–91) vs. 6 days (IQ: 4–11), *p* = 0.011].

In period A, molecular studies were performed in three patients with galactosemia, one with argininemia (29), and one with MacCune-Albright syndrome (30), previously reported. In period B, molecular studies were performed in patients with Alagille syndrome and some metabolic diseases (27).

Additionally, since the NGS panel became available, we applied it, so far, to 13 patients of this series (see details in Table 4) (31–36). We highlight a pair of siblings (brother and sister), diagnosed with neonatal sclerosing cholangitis in period A, based on clinical, biochemical, imaging, and histological criteria (26). NGS panel in the sister (the brother declined testing) allowed us to identify the genetic basis of their disease (case 1).

**Table 4:** Results from NGS panel in 13 patients.

**ID**	**Gender**	**Birth year**	**Liver clinical phenotype/diagnosis**	**Outcome**	**Genes**	**Variants**	**Interpretation**
1	F	1986	CLD *Neonatal sclerosing cholangitis*	Alive OLT	86	***DCDC2*** (NM_001195610.1)—c.942del [p. (Gly315Glufs*32)]—in Intron 9 and Exon 19, in HOMOZYGOSITY	**A variant not previously described, leading to a premature STOP codon, most likely pathogenic**. In this gene were previously described other pathogenic variants in association with **neonatal sclerosing cholangitis** (AR), with deafness type 66 and **nephronophthisis type 19**.
2	F	1992	CLD *Argininemia + a second disease?* (pruritus + dislipidemia)	Alive OLT	86	***ARG1*** (NM_001244438.1)—c.61C>T [p.(Arg21*)]—Exon 2, in HOMOZYGOSITY	A variant in homozygosity, previously associated with argininemia (31). **No second disease confirmed**.
3	M	1998	Liver failure	Alive Cured	54	No variants potentially pathogenic	No underlying disease confirmed.
4	F	2002	Liver failure	Alive Cured	54	No variants potentially pathogenic	No underlying disease confirmed.
5	M	2009	*Transient cholestasis* (without risk factors)	Alive Cured	54	***DCDC2*** (NM_001195610.1)—c.1283A>T [p. (Asp428Val)]—Exon 10, in HETEROZIGOSITY***DGUOK*** (NM_080916.2)—c.4G>T [p. (Ala2Ser)]—Exon 1, in HETEROZIGOSITY***VP5338*** (NM_018668.4)—c.1148T>A [p.(lle383Asn)]—Exon 15, in HETEROZIGOSITY	**Three variants in heterozygosity, all of unknown clinical significance**. The bioinformatic analysis indicates that the first two will be benign, and the third (not previously described) will be pathogenic.
6	M	2011	CLD *Idiopathic cholestasis* (with raised total bile acids; no improvement with UDCA)	Alive CLD	54	***CYP781*** (NM_004820.3)—c.928C>T [p. (Arg310Trp)]—Exon 4, in HETEROZIGOSITY	**A variant of unknown clinical significance**, whose bioinformatic analysis indicates that it will be pathogenic. Both progenitors have normal liver tests; the father has raised total bile acids; genetic tests of both parents are in course.
7	F	2015	CLD *Biliary atresia + a second disease?* (microcephaly + cognitive delay + enteropathy)	Alive OLT	54	***ATP8B1*** (NM_005603.4)—c.607A>G [p. (Lys203Glu)]—Exon 7 in HETEROZYGOSITY	A rare variant (rs56355310) of **unknown clinical significance**. There are pathogenic variants reported in this gene in patients with PFIC-1 (AR). Heterozygosity was described in association with transient cholestasis (32).
8	M	2016	*Transient cholestasis* (without risk factors) *Alagille Syndrome?* (facies + embriotoxon)	Alive ALT elevated	95	***NOTCH2*** (NM_024408.3)—c.7223T>A [p. (Leu2408His)]—Exon 34 in HETEROZYGOSITY	**A variant** formerly identified by Sanger sequencing of the gene, classified as of unknown clinical significance and considered as potentially pathogenic, is **currently seen as benign**. The parents refused to do the genetic test.
9	M	2016	*Transient cholestasis* (without risk factors)	Alive Cured	54	***ABCB11*** (NM_003742.2)—c.1460G>A [p. (Arg487His)]—Exon 14, in HETEROZIGOSITY	A variant in heterozygosity, **previously associated with PFIC-2** (33).
10	M	2016	*Transient cholestasis* (without risk factors)	Alive Cured	54	No variants potentially pathogenic	No underlying disease confirmed.
11	F	2018	CLD *Deficit alfa-1-AT (ZZ) + a second disease?* (pale stools)	Alive CLD	54	***SERPINA1*** (NM_ 001127701.1)—c.1096G>A [p. [Glu366Lys)]—Exon 7, in HOMOZIGOSITY	A variant in homozygosity, previously associated with deficit of alpha-1-antitrypsin (34). **No second disease confirmed**.
12	M	2018	*Transient cholestasis* (with UTI *E. coli*)	Alive Cured	86	No variants potentially pathogenic	No underlying disease confirmed.
13	M	2019	*Transient cholestasis)* (with starvation)	Alive Cured	86	***CFTR*** (NM_000492.3)—c.1210-11T>G (r.?)—Intron 9, in HETEROZIGOSITY***CFTR*** (NM_000492.3)—c.2991G>C [p.(Leuc997Phe)]—Exon 19, in HETEROZIGOSITY	Both variants are currently classified as variants of **unknown clinical significance**. The first was previously described as responsible for primary ciliary dyskinesia (AR) (35). The second was previously described as responsible for congenital bilateral absence of the vas deferens (AR) (36).

*CLD, chronic liver disease; OLT, orthotopic liver transplant; UTI, urinary tract infection; AD, autosomal dominant; AR, autosomal recessive*.

*Here the classification of “Transient NC” is applied by the authors only in accordance with the evolution of liver clinical phenotype*.

### Treatment

Kasai's surgery was performed at a significantly younger age over time (*p* = 0.002) (Table 2), but the success of this surgery did not increase significantly after the year 2000 (22 vs. 60%), *p* = 0.072).

Treatment with UDCA, administered to patients with A1ATD was not associated with a significantly better outcome (55 vs. 83%, *p* = 0.111). Four out of the 23 treated patients had unfavorable outcome, in comparison with 5 out of 11 untreated patients; no side effects were observed.

OLTs were significantly more frequent in period A (*p* = 0.014). Differences in relation to the median age at which they were performed were not significant. In both periods, OLT performance varied significantly according to the underlying entity. In period A, OLT was performed in 6 out of 9 patients with biliary atresia, 6 out of 25 with A1ATD, 2 out of 2 with neonatal sclerosing cholangitis, 2 out of 2 with tyrosinemia, 1 out of 1 with argininemia, and 1 out of 3 with Alagille syndrome (*p* = 0.035). In period B, OLT was performed in 8 out of 15 patients with biliary atresia, and in 2 out of 9 with A1ATD (*p* = 0,020).

### Outcome, Morbidity, and Mortality

Outcome was unfavorable in 47 patients (31%), without significative difference over time. Co-morbidities were identified in 26% (*n* = 40) and death occurred in 12% (*n* = 18), both not significantly different over time (Table 5). Early death rate (<12 months-old) occurred in 7% (*n* = 10; 55% of all mortality), from which 7 patients had metabolic or infectious diseases or idiopathic cholestasis—*the fast killers*. Mortality was not significantly different between genders.

**Table 5:** Outcome, morbidity and mortality: comparison between periods A and B.

	**Cohort (*****N*** **=** **154)**		**Before year 2000 (A)**		**After year 2000 (B)**		*p*-value^*^
	***N***	**(%)**	***N***	**(%)**	***N***	**(%)**	
**OUTCOME**							0.108
Favorable	107	(70)	42	(63)	65	(75)	
*Biliary diseases (n = 34)*							0.171
Favorable	14	(41)	4	(27)	10	(53)	
*Biliary atresia (n = 24)*							0.191
Favorable	7	(2)	1	(11)	6	(40)	
*A1ATD (n = 34)*							>0.999
Favorable	25	(4)	18	(72)	7	(78)	
*Infectious diseases (n = 20)*							0.400
Favorable	19	(95)	7	(88)	12	(100)	
*Metabolic diseases (n = 18)*							0,664
Favorable	10	(56)	5	(63)	5	(50)	
*Transient cholestasis (n* = *24)*							-
Favorable	24	(100)	4	(100)	20	(100)	
*Other diseases (n* = *12)*							>0.999
Favorable	8	(67)	3	(75)	5	(63)	
*Idiopathic (n* = *12)*							0.523
Favorable	7	(58)	1	(33)	6	(67)	
**Status of liver disease at present**							0.125
Cure	86	(56)	35	(52)	51	(59)	
CLD without cirrhosis/PTH	21	(14)	7	(10)	14	(16)	
CLD with cirrhosis/PTH	3	(2)	0	(0)	3	(3)	
OLT	26	(17)	16	(24)	10	(12)	
Death	18	(12)	9	(13)	9	(10)	
**CO-MORBIDITIES**							0.881
Yes	40	(26)	17	(25)	23	(26)	
Brain	17	(11)	7	(10)	10	(12)	
Eyes	1	(.6)	1	(2)	0	(0)	
Ears	1	(.6)	1	(2)	0	(0)	
Others	11	(7)	6	(9)	5	(6)	
Brain and others	8	(5)	0	(0)	8	(9)	
Brain and eyes	2	(1)	2	(3)	0	(0)	
**MORTALITY**							0.554
Yes	18	(12)	9	(13)	9	(10)	
*Early death (<12 months old)*	10	(7)	4	(6)	6	(7)	0.817
*Cause of death*							0.959
Liver disease	8	(44)	4	(44)	4	(44)	
Other organs related to UE	7	(39)	3	(33)	4	(44)	
Other disease	3	(17)	2	(22)	1	(11)	

* *Chi-square test; A1ATD, alpha-1-antitrypsin deficiency; CLD, chronic liver disease; PTH, portal hypertension; OLT, orthotopic liver transplantation; UE, underlying entity*.

Mortality details were as follows: 9 patients died in period A (biliary atresia *n* = 3, Caroli disease *n* = 1, A1ATD *n* = 1, Alagille syndrome *n* = 1, Herpes infection *n* = 1, idiopathic cholestasis *n* = 2); from those, 5 deaths were potentially avoidable: 3 died while waiting for OLT (2 with biliary atresia and 1 with neonatal idiopathic hepatitis), 1 with Caroli disease died from sepsis, and one with A1ATD died from CMV infection; additionally, 2 died after OLT complications. The same number of patients died in period B (mitochondrial disorders *n* = 3, Zellweger syndrome *n* = 1, Menkes disease *n* = 1, Short-bowel syndrome *n* = 1, idiopathic cholestasis *n* = 2), but these were unavoidable deaths and none died after OLT.

**Figure 2:**
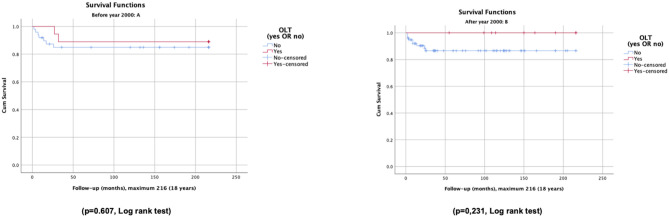
Overall survival and survival without OLT of patients with neonatal cholestasis, before (A) and after (B) the year 2000.

Overall survival and survival without OLT had no significant improvement during the period of observation (in periods A and B, 86 vs. 88%, and 85 vs. 87%, respectively). However, in period B, with OLT we achieved the goal of 100% of survival rate. Survival rates of patients with and without OLT are disclosed in Figure 2.

**Figure 3:**
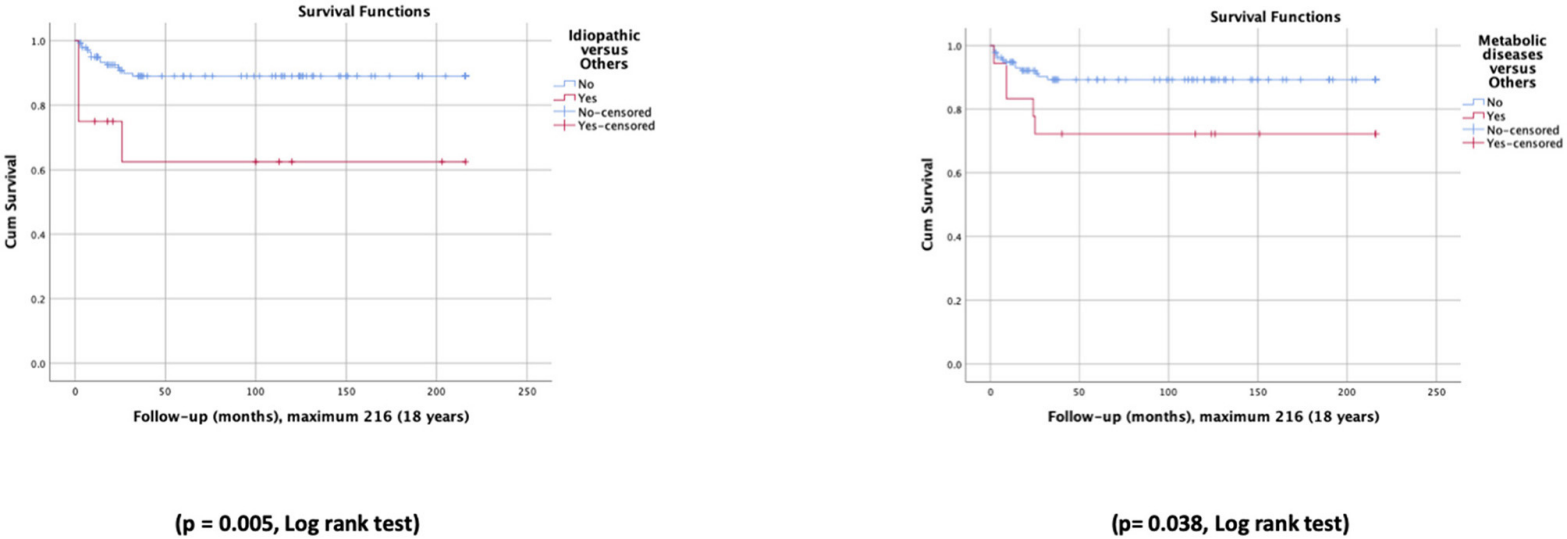
Survival curves for patients with idiopathic cholestasis vs. other patients, and metabolic diseases vs. other patients.

Survival rates were different according to subgroups of underlying entities. The idiopathic cholestasis subgroup exhibited significantly lower overall survival rates than other patients (63 vs. 89%) as well as the metabolic diseases subgroup (72 vs. 89%) (Figure 3). The survival rate in patients with biliary atresia, with and without OLT, was not significantly different (93 vs. 75%); the same happened with patients with A1ATD (100 vs. 96%).

## Discussion

NC is the form of expression of many types of liver injury with a myriad of underlying entities (15). Geographic variability is explained by genetic and environmental factors, and also depends on the expertise of the healthcare services and their technical resources. Over time, advances in diagnostic tools has changed the epidemiological landscape of this entity.

Biliary atresia is the most common cause of NC (15). A1ATD was the first genetic disease to be reported presenting with NC and progressing to liver cirrhosis (37) and played an important role in some European populations, namely in England (38) and Sweden (39). However, this may no longer be the case in England taking into account the different results obtained by Humphrey et al. (13) vs. Mieli-Vergani et al. (38). In our series, A1ATD was overall more frequent than biliary atresia, with a slightly higher rate than what had been observed in English and Swedish studies, and much higher than the rate of 4% described in the systematic review (15). The prominence of this specific condition in our cohort may be explained by the high prevalence of the disease in Northern Portugal (40), a region known for its Celtic genetic heritage. Noteworthy, we have seen a prevalence decrease over the past two decades. Several reasons can explain the lower number of cases: decrease in birth rate, greater diagnostic capacity in secondary healthcare facilities, better outcome due to early diagnosis and better supportive treatment, and our availability for external consultancy, all together avoiding the referral. In parallel with the loss of predominance of A1ATD after the year 2000, we observed the arrival and rise of the “new kid on the block”—the transient cholestasis—surpassing the “old players.”

After the year 2000, several studies reported an emergent subset of patients, denominated as “transient,” “prematurity-associated,” “TPN-associated,” or “sepsis-associated” (15). This new subset of patients shares a higher level of exposure to risk factors (19, 41). Their role as determinants or co-factors of NC is far from being totally clear. In our study, one third of the other patients also had risk factors. Interestingly, the biliary atresia patients had significantly less risk factors. This is in contrast with the A1ATD patients in which the pathophysiology of the liver disease (42), may be affected by the risk factors, and so the prevention or treatment of those may also be contributing to the downtrend in prevalence. On the other hand, the increase in transient cholestasis may be explained by the increased rate of prematurity and survival of premature and sick newborns, and an increase in referrals of these to tertiary centers. Previous studies (17, 18) did not address underlying entities as possible determinants or cofactors of cholestasis in patients with transient cholestasis, but in our study we have verified that in out of 9 patients referred with this diagnosis we could identify both sporadic (e.g., CMV infection, choledochal cyst) and genetic etiologies (e.g., A1ATD). Finally, identification of etiological diagnosis might have been limited due to insufficient diagnostic tools. The assumption of the diagnosis of transient cholestasis is one of exclusion and the current guidelines for diagnostic approach do not fully address this problem (8). In clinical practice the current main question is: “*how far should we investigate these patients for underlying entities?”*

The prevalence of idiopathic cases has decreased significantly according to a recent European study (11). In our center, the prevalence rate has not decreased over time—as a matter of fact, we obtained similar figures to the ones reported by Hoerning et al. Improved detection rate is likely to be attained with the availability of new molecular technologies. Recently, Nicastro et al. (43) developed a prospective study that included 125 patients, of which 50 subjects underwent a through diagnostic protocol that included genetic testing—and they obtained a detection rate of 60% which is much higher than reported in previous studies (44). Our preliminary data supports the perspective that patients that remain undiagnosed despite standard of care diagnostic tools may benefit from NGS panels for further clarification. Neonatal sclerosing cholangitis is the poster child of this paradigm as it allowed to establish a genetic basis and point to the possible pathophysiology—a novel liver-based non-motile ciliopathy, sometimes associated to nephronophthisis (45, 46); which our study also supports since we describe a novel variant. Still, despite the advances that new molecular technologies brought to this field, a word of caution is warranted as genetic studies can be misleading (e.g., case 8, Alagille syndrome, later unconfirmed), of difficult interpretation (e.g., case 5, co-existence of 3 variants in heterozygosity) or raise more questions instead of simply giving us answers (e.g., when parental testing is required for re-classification of variants which comes at increased costs). We did not offer NGS panel testing to patients with transient cholestasis but we did offer it to patients with transiently evolving cholestasis without risk factors, mainly searching for some treatable diseases such as Niemman-Pick type C (47) or citrin deficiency (48), and we had some surprising results requiring further clarification.

According to Feldman and Sokol (7), genetic evaluation will necessarily change the diagnostic paradigm and give rise to current and emerging diagnosis algorithms that will certainly include this tool. From our experience, NGS panel should be used with caution and in selected cohorts. As we recently stated elsewhere (27), we believe that this tool must not be used as first-line, and should definitely not be used in the absence of skilled clinical guidance. Therefore, it does not make sense to talk about a paradigm shift, since any decision algorithm must always have clinical and biochemical markers. Additional work is needed to formally assess the cost-effectiveness of these studies and to understand what is their ideal place in the algorithm tree. In Portugal, we still need to improve on NGS panel availability and turn-around times that allow for diagnosis in a timely manner. We propose its use as previously described by Moreira da Silva et al. (27). In clinical practice we do not recommend transient cholestasis patients to be offered NGS panels. However, including these patients in research studies will be useful as they might contribute to a better understanding of the pathophysiology of NC. We highlight the fact that no positive genetic test will ever subside one of our greatest sources of anxiety—the exclusion of biliary atresia—since it can coexist with other entities.

Despite all the advances, NC remains globally associated with high mortality and morbidity (14). Our data is in line with this observation, as one third of the patients had an unfavorable outcome. In addition, a significant number of patients with “favorable outcome” presented mild chronic liver disease and their long-term outcome (adulthood) is uncertain. Mortality and morbidity rates are difficult to compare, due to the different follow-up periods in each study. However, the comparison of our findings with the study by Hoerning et al. (11) showed a similar mortality rate, but, contrary to this study, which found a higher mortality rate in biliary atresia (before and after OLT), in our study, metabolic diseases and idiopathic cases were the main responsible for mortality.

As far as we know, there are no publications with long-term survival studies, except in the subgroup of biliary atresia that has been well-studied. In biliary atresia, first the Kasai surgery (49, 50), then OLT (51, 52), and, more recently, in some countries the centralization of diagnosis and treatment (5, 6), and in others, universal newborn screening programs (53), produced advances with impact on overall survival and survival with native liver (5, 54). Our study presents the survival curves of a cohort of 154 patients with NC for a period of nearly 35 years, namely overall survival and survival without OLT, as well as the comparison of these curves before and after the year 2000. We concluded that outcomes and survival did not improve significantly over time, and the number of patients who survived due to the benefits of OLT was not significant. Outcomes and survival varied with the underlying entities. In our study, patients with biliary atresia had long-term overall survival similar to other patients, and survival rates without OLT were not significantly lower. Metabolic diseases and idiopathic cholestasis showed significantly shorter survival curves than those of other patients.

Early recognition of cholestasis is fundamental to attain a better outcome (55). In our cohort, early recognition of cholestasis improved significantly over time. This was due to a continuous investment in the postgraduate training of pediatricians and pediatric nurses—in a pilot study developed locally that entailed a survey and photographs of normal and pale stools, these healthcare professionals obtained the best results (3). However, there is still room for improvement, especially at the level of primary healthcare services. Taking in to account the high morbidity and mortality, a universal screening program for neonatal cholestasis would be very useful; however, there are numerous underlying entities expressing at variable timings, which makes screening a very complex task. So far, only one screening method has been developed successfully: the stool color card that screens for biliary atresia. This screening tool as not been adopted in our country yet—but could easily be integrated into our Health Surveillance program of the Child and Adolescent. There are some promising studies using serum levels of conjugated bilirubin in the first days of life (56, 57) as a screening tool; these studies, if validated in larger populations, could point toward a screening method for other underlying entities in addition to biliary atresia. In our country, this parameter can also be easily added to the existing endocrine-metabolic screening program (23).

Other factors may interfere with the outcome of patients, such as the accuracy in the diagnosis of the underlying entities and the availability of specific medical and surgical treatments (10). In our center, patients had access to modern diagnostic techniques, as well as medical and surgical therapies according to international guidelines and to our institutional resources over time. After the year 2000, patients benefited from a qualified and stable surgical team, but the improvement in the success rate of Kasai surgery was not significant. Given the low number of cases/year, and according to data from other countries (6), since April 2019, the patients managed in our center were referred to perform Kasai surgery at the only pediatric liver transplant center in Portugal.

In summary, our data suggest that transient cholestasis became a very important etiology of NC, it can hide underlying entities, and requires specific guidance. The challenge on to what extent pursuing an etiological diagnosis still remains. The role played by risk factors in NC is far from clear. NGS panels can provide important inputs on disease diagnosis, but if applied without strict criteria and expertise they can open a Pandora's box due to misinterpretation. Despite all the advances in accurate diagnosis and timely management—including early recognition of cholestasis—the improvement in patient outcomes and survival were still not significant.

## Data Availability Statement

The data analyzed in this study is subject to the following licenses/restrictions: The data are recorded in the patients' clinical files and in the hospital databases. In order to have access, it is necessary to ask for authorization from the Ethics Committee and the Board of Directors of the hospital. This authorization was requested and obtained to carry out this study. Requests to access these datasets should be directed to Ethics Committee, secretariado.etica@chporto.min-saude.pt, and Departement of Education, Training and Research (DEFI), secretariado.cg.defi@chporto.min-saude.pt.

## Ethics Statement

This study, involving human participants, was analyzed and approved by the Ethics Committee (Comissão de Ética para a Saúde, CES) and by the Research Coordinating Office of the Department of Education, Training, and Research (Gabinete Coordenador da Investigação do Departamento de Educação, Formação, e Investigação, DEFI) and afterwards reviewed and approved by the board of administration (Conselho de Administração) of our hospital (Centro Hospitalar e Universitário do Porto, Portugal).

## Author Contributions

ES diagnosed and followed patients, designed the study, collected and analyzed data, and elaborated the draft of the manuscript. AA and SF diagnosed and followed patients, created databases, and collected and analyzed data. EM diagnosed and followed patients and collected data. MV analyzed data and critically reviewed the manuscript. AS-S and AL critically reviewed the manuscript. All authors contributed to the article and approved the submitted version.

## Conflict of Interest

The authors declare that the research was conducted in the absence of any commercial or financial relationships that could be construed as a potential conflict of interest.
